# Relationship between knowledge on COVID-19 and psychological distress among students living in quarantine: an email survey

**DOI:** 10.3934/publichealth.2021007

**Published:** 2021-01-12

**Authors:** Areeb Khalid, Muhammad Waqar Younas, Hashim Khan, Muhammad Sarfraz Khan, Abdur Rehman Malik, Adam Umair Ashraf Butt, Basit Ali

**Affiliations:** 1 MBBS, Rawalpindi Medical University, Pakistan; 2 Benazir Bhutto Hospital, Rawalpindi, Pakistan

**Keywords:** psychological distress, the Kessler-10 (K10), COVID-19, quarantine

## Abstract

Psychological distress is a generic term which refers to “feeling of emotional strain” that affects our normal mental and physical functioning. The aim of this study is to investigate the psychological distress perceived by the Pakistani students living in quarantine and to determine risk and protective factors, including knowledge of COVD-19, among this population. It is a descriptive cross-sectional study conducted from February to May 2020. Students enrolled at different colleges and universities of Pakistan participated in this survey. One-way analysis of variance (ANOVA) is computed for comparing knowledge scores of participants having different levels of psychological distress. A total of 937 participants completed the survey questionnaire, with slightly more male respondents (60.6%) than female (39.4%). The average age of survey participants is 22.0 years (SD = 3.01), with majority (76.2%) belonging to urban areas. The mean COVID-19 knowledge score is 8.91 (SD = 1.69, range: 1–12), suggesting an overall 74.25% precision rate for this knowledge test for individual participant. The participants scored least knowledge regarding the disease transmission, showing a percentage correctness of only 40%. Majority of the participants (57.3%) are likely to be well, while others (42.7%) have shown symptoms of mental distress. The analysis reveals that participants with moderate mental distress (M = 8.81, SD = 2.37) and those with severe mental distress (M = 8.75, SD = 2.69) scored lower than participants who were likely to be well (M = 9.49, SD = 1.71). Our study concludes that a higher knowledge base regarding the disease will help to mitigate distress levels. Our study suggests that in order to deal with this pandemic effectively, the knowledge regarding COVID-19 should be properly conveyed to general public. It is need of the hour to address mental issues of the population aggressively along with providing awareness about COVID-19.

## 1. Introduction

The 2019 Novel Coronavirus (2019-nCoV) that emerged in December 2019 in Wuhan, China, quickly spread worldwide. The World Health Organization (WHO) named this disease as Coronavirus Disease 2019 (COVID-19). Due to its rapid spread, WHO declared it a Public Health Emergency of International Concern on 30^th^ January 2020 [1]. By this time, The SARS-CoV-2 had been spread to more than 20 countries [2]. The rapid outbreak of COVID-19 from China to the rest of the world was alarming. On 11 March 2020, The World Health Organization (WHO) officially declared the COVID-19 outbreak a global pandemic [3].

In Pakistan, the first case of COVID-19 was reported in Karachi on 26^th^ February 2020 [4]. Upon its arrival, main focus of Pakistan Government was on quarantine and self-isolation of infected as well as suspected people [5]. To minimize COVID-19 spread, quarantine, along with other preventions and control measures such as self-isolation, social distancing, and hand hygiene, are considered to be most effective [6]. Previous studies on similar topics such as SARS (severe acute respiratory syndrome) and MERS (Middle East respiratory syndrome) indicate that, although quarantine has been used successfully for centuries to contain infectious diseases, the benefits of quarantine need to be weighed carefully against its psychological effects [7]. People in quarantine have a tendency to develop lots of psychological issues. They are at high risk of developing psychological distress, fear, and risk perceptions. This psychological distress can impart prolonged effects on emotional and physical health of people. It is mainly due to the lack of knowledge or ambiguous information that is common in the initial period of disease outbreaks [8].

Psychological distress is a general term used to describe unpleasant feelings or emotions that impact your level of functioning [9]. A thorough review of literature on previous epidemics and pandemics (e.g. SARS, MERS, H1N1 etc.) shows that psychological distress and fear have been observed in people due to lack of knowledge about that disease. This distress has ultimately resulted in numerous deaths, panic, and economic losses [8],[10]. Furthermore, certain temperaments and attachment styles are proven to be a risk factor for an increased level of psychological distress during the current pandemic [11].

Pakistan has a collectivistic culture where people are more dependent on socialization (social support and social connectedness). Despite the strict advice of experts to observe social distancing, people struggle to comprehend the ruthlessness of COVID-19. This has induced panic and mental distress in people rather than just keeping themselves secluded and isolated to deal with this pandemic. The psychological impact of quarantine has been shown to include confusion and distress in previous studies as well [12].

Consequently, it is important to find out factors that could contribute to the development of psychological distress among people. In this regard, a previous study has shown that fear of COVID-19 is related to Intolerance of Uncertainty [13]. This could suggest that having insufficient knowledge/information regarding COVID-19 could lead to uncertainty and thus fear of COVID-19. A study conducted in India has shown a moderate level of knowledge about the COVID-19 infection but an adequate knowledge about its prevention in the population [14]. Therefore, as the majority of population lacks in sufficient knowledge about the disease, it may result in an increased level of panic and psychological distress. Thus, we are focusing on impact of awareness/unawareness about the disease on the level of psychological distress among the population.

This psychological distress among the quarantined population in Pakistan is needed to be analyzed. Accordingly, our study is aimed to investigate the psychological distress perceived by the Pakistani students living in quarantine and to determine risk and protective factors, including knowledge of COVD-19, among this population. Based on the previous discussion, we did a descriptive cross-sectional study from February to May 2020. We developed a COVID-19 knowledge questionnaire having 12 questions. It was in accordance with WHO guidelines for clinical and community management of COVID-19 [15]. Psychological distress was measured using the Kessler-10 (K10). We hypothesized that a lack of knowledge and awareness of COVID-19 would be associated with higher levels of psychological distress. Thus, our study emphasized on the need to deliver insight and awareness to the people about COVID-19 and its prevention to decrease the prevalence of psychological distress in our society during this pandemic.

## 2. Materials and methods

### 2.1. Study design

This study was conducted using a descriptive cross-sectional design.

### 2.2. Study participants

Students enrolled at different colleges and universities of Pakistan participated in this survey. Incomplete forms were excluded from the study. Out of 1500 forms distributed, 937 were completely filled out, giving a correct-response rate of 62.77%. All participants were informed about the survey objectives. The confidentiality of all participants was completely maintained.

### 2.3. Data collection

With the collaboration of a non-profitable welfare organization named Solidarity among Young Nation for Change (S*YNCH*), we sent invitations through email using simple random sampling technique to students. This study was conducted from February to May 2020.

### 2.4. Measures

According to guidelines for clinical and community management of COVID-19 by WHO, a COVID-19 knowledge questionnaire was developed by the authors [15]. The questionnaire had 12 questions; 4 regarding clinical presentations (K1–K4), 3 regarding transmission routes (K5–K7), and 5 regarding prevention and control (K8–K12) of COVID-19. These questions were answered on a true/false basis with an additional “I don't know” option. A correct answer was assigned one point, and an incorrect/unknown answer was awarded zero points. The total knowledge score ranged from 0 to 12, with a higher score denoting a better knowledge of COVID-19. The Cronbach's alpha coefficient of the knowledge questionnaire was 0.647 in our sample, indicating acceptable internal consistency.

Psychological distress was measured using the Kessler-10 (K10). This 10-item, self-administered questionnaire was developed for use in the USA National Health Interview Survey [16]. The K10 was kept in its English version. There was no need for translating it into local language because primary medium of teaching/instruction of students under study was English. It is designed to yield a global measure of psychological distress based on questions related to anxiety and depressive symptoms experienced in the most recent 30-day period. Each item is presented in a 5-point Likert scale format, with responses ranging from “none of the time” to “all of the time”. The sum of these ten items produces a combined score out of a possible 50, where higher scores indicate more considerable psychological distress [16],[17]. K10 scores were further divided into similar levels of psychological distress defined as a score under 20 are likely to be well, score 20–24 are likely to have a mild mental distress, score 25–29 are likely to have a moderate mental distress and score 30 and over are likely to have a severe mental distress. The Cronbach's alpha value calculated for this questionnaire was 0.895, indicating acceptable internal consistency.

A sociodemographic questionnaire consisting of age, gender, city, and level of education was used. People were also asked about having any family member suffering from the coronavirus.

### 2.5. Statistical analysis

Frequencies and percentages of correct answers and demographic details were described. The Spearman correlation coefficient was calculated to find the strength of the relation between knowledge scores and demographic variables. One-way analysis of variance (ANOVA) comparing the knowledge scores of participants having different levels of psychological distress were computed. In all statistical analyses, *P*-values less than 0.05 were accepted as statistically significant. Data were analyzed using the Statistical Package for Social Sciences (SPSS) v.23.0 (IBM, Armonk, U.S.).

## 3. Results

A total of 937 participants completed the survey questionnaire. Among them, the average age was 22.0 years (SD = 3.01). Out of 937,568 (60.6%) were male and 369 (39.4%) were female. Table 1 shows the demographic distribution.

**Table 1.** Sociodemographic characteristics of participants (N = 937).

Characteristics	Values	
	N	%
Age(years)		
≤20	295	31.5%
21–25	569	60.7%
≥26	73	7.8%
City		
Rural	223	23.8%
Urban	714	76.2%
Educational status		
HSSC/A-levels	122	13.0%
Under-graduate	611	65.2%
Graduate	138	14.7%
Doctor of Philosophy (Ph.D.)	66	7.0%
COVID-19 positive Family member		
Yes	70	7.5%
No	867	92.5%

Note: N = no. of observations; HSSC: Higher Secondary School Certificate (HSSC); A-levels: Advanced level qualification.

### 3.1. Knowledge scores

The correct answer rates of the 12 questions on the COVID-19 knowledge questionnaire were 11.1% to 96.6%. The mean COVID-19 knowledge correctness score for each individual participant was 8.91 (SD = 1.69, range: 1–12), suggesting an overall 74.25% (8.91/12*100) percentage correctness on this knowledge test. The participants scored the least knowledge regarding the disease transmission, showing percentage correctness of only 40%. Table 2 shows component wise percentage correctness of the total sample.

**Table 2.** Percentage correctness regarding COVID-19 knowledge questionnaire (N = 937).

Questions	Yes	No	I don't know	Percentage correctness of the total sample
Clinical presentation knowledge				76.53^a^%
K1. The main clinical symptoms of COVID-19 are fever, fatigue, dry cough, and myalgia.	91.2%	4.1%	4.7%	
K2. Unlike the common cold, stuffy nose, runny nose, and sneezing are less common in persons infected with the COVID-19 virus.	54.2%	22.5%	23.3%	
K3. There currently is no effective cure for COVID-19, but early symptomatic and supportive treatment can help most patients recover from the infection.	85.3%	4.8%	9.9%	
K4. Not all persons with COVID-19 will develop to severe cases. Only those who are elderly, have chronic illnesses, and are obese are more likely to be severe cases.	75.4%	16%	8.6%	
Knowledge regarding transmission routes				40^a^%
K5. Eating or contacting wild animals would result in the infection by the COVID-19 virus.	48%	27.8%	24.2%	
K6. Persons with COVID-19 cannot infect the virus to others when a fever is not present.	11.1%	73.3%	15.6%	
K7. The COVID-19 virus spreads via respiratory droplets of infected individuals.	81.1%	7.7%	11.2%	
Knowledge regarding prevention and control				89.92^a^%
K8. Ordinary residents can wear general medical masks to prevent the infection by the COVID-19 virus.	80.8%	12.2%	7.0%	
K9. It is not necessary for children and young adults to take measures to prevent the infection by the COVID-19 virus.	15.4%	81.9%	2.8%	
K10. To prevent the infection by COVID-19, individuals should avoid going to crowded places such as train stations and avoid taking public transportations.	96.6%	1.6%	1.8%	
K11. Isolation and treatment of people who are infected with the COVID-19 virus are effective ways to reduce the spread of the virus.	95.7%	1.7%	2.6%	
K12. People who have contact with someone infected with the COVID-19 virus should be immediately isolated in a proper place. In general, the observation period is 14 days.	94.6%	1.7%	3.7%	
Total average percentage correctness				68.82^a^%

Note: a presents average percentage correction; bold font indicates correct answers.

### 3.2. Psychological distress

The majority of the participants, 537(57.3%), were likely to be well, while 95 (10.1%) were likely to be suffering from a severe mental distress. Table no.3 shows the gender-wise distribution of psychological distress levels.

**Table 3.** Gender wise distribution of psychological distress levels.

Psychological distress levels	Male		Female		Total	
	N	%	N	%	N	%
Mildly unwell	113	19.9%	79	21.4%	192	20.5%
Moderately unwell	63	11.1%	50	13.6%	113	12.1%
Severely unwell	40	7%	55	14.9%	95	10.1%

ANOVA, comparing the knowledge scores of participants having different levels of psychological distress, was computed. A significant difference was found among the participants with different distress levels (F (3,933) = 8.52, p < 0.001). Tukey's HSD was used to determine the nature of the differences between the instructors. This analysis revealed that participants who were likely to have a moderate mental distress (M = 8.81, SD = 2.37) and those who were likely to have a severe mental distress (M = 8.75, SD = 2.69) scored lower than participants who were likely to be well (M = 9.49, SD = 1.71). Participants who were likely to have a mild mental distress (M = 9.62, SD = 1.64) were significantly different from those who were likely to be severely unwell and mildly unwell but not significantly different from those who were well. In summary, our results suggest that participants who were likely to be unwell; they have lower knowledge scores. However, it should be noted that they should be moderately or severely unwell to see an effect.

## 4. Discussion

Epidemics and pandemics are periodic phenomena. Lack of awareness and knowledge among the general population may lead to increase level of psychological distress. As of yet, there have been limited number of studies addressing the distress caused by COVID-19. However, the situation is still developing and we aim to add our contribution to the growing literature on this topic. Our study has shown some promising results and has given a comprehensive outlook on the distress among the population and their level of knowledge regarding the COVID-19 pandemic.

A similar study has been done in India regarding knowledge, attitude, anxiety & perceived mental healthcare need in the Indian population during the COVID-19 pandemic. They conducted an online survey on the people facing the pandemic [14]. Similarly, another research was conducted in the United Kingdom regarding H1N1 [18]. They held a telephonic survey over four days in the native population among those who can speak English. Both the COVID-19 and the H1N1 pandemics are similar in origin and cause respiratory illness spreading through respiratory droplets.

Our study found a significant positive correlation between knowledge scores and educational status i.e. participants with a higher education level showed higher knowledge scores. A survey carried out in Hong Kong [19], as well as one in Qatar [20] during the SARS outbreak, shows similar results. On the contrary, a study by Ilesanmi et al., regarding the Ebola virus in Nigeria, found conflicting results. A possible reason for this is the limited sample population of the study, which primarily focused secondary school students [21].

Secondly, there was no significant relationship between age group, gender, and knowledge scores, while a significant negative correlation was found between knowledge and family members who are COVID-19 positive (p < 0.05). This result is self-explanatory as a lack of knowledge about the disease and its prevention etc. was most possibly the cause of infection.

Regarding the specific areas of knowledge, the highest score was found for prevention and control (89.92%), while the lowest was for the mode of transmission (40%). The score for the clinical presentation of the disease was found to be 76.53%. This is per the research carried out in India during the COVID-19 pandemic, which states adequate knowledge among the population regarding preventive measures [14]. However, following the H1N1 epidemic, results found by Johnson et al. showed that a considerable amount of general population lacked awareness of measures for the prevention of the pandemic in 2016 [22]. We believe that due to the serious situation of this pandemic and the excessive news reports on this public health emergency, people would actively learn knowledge for prevention and control of the infectious disease. However, more awareness regarding prevention and control against COVID-19 has been found compared to the route of transmission and clinical presentation of the disease, respectively. It was possibly due to the government and media emphasizing more on the preventive measures. Least knowledge for disease transmission is probably due to the pandemic's ambiguous condition and the fact that we are still learning about the disease as the situation progresses.

Similar to our results, Everts et al. found that the H1N1 pandemic caused significant anxiety among the public during 2009–10 [23]. Our study also gives considerable evidence that psychological distress was more significant in females than in males. A study in China by Wang et al. showed that women suffered from mental distress and stress, anxiety, and depression during the initial stages of the COVID-19 epidemic [24]. This is consistent with a survey carried out in Iran during the current COVID-19 outbreak [25].

A possible reason for the anxiety and distress among the population may be widespread fear-mongering by the media regarding COVID-19. According to Roy et al., the media influences the mental well-being of the general population and may add to anxiety [14]. At the same time, increased exposure may lead to higher levels of distress among the population [26]. Similarly, more anxiety among people hearing more news about COVID-19 is also found by WHO and Moghanibashiet et al. [27],[25].

Our study has shown that a higher knowledge score corresponded to a lower level of distress among the population. However, for this relationship to be significant, the person had to be suffering from a moderate or severe distress. The knowledge scores of the participants who were well or were suffering from only mild levels of stress were not significantly different. Thus, this shows that a higher level of knowledge alleviates the amount of psychological distress caused by the ongoing pandemic. Possible factors that could contribute to psychological distress in the general population during the COVID-19 pandemic may include; gender, social support, specific experiences with COVID-19 infection, length of isolation, and amount of exposure to the media [26].

There were few limitations in our study. First and foremost, our study was limited to people who had access to a smart-phone and email ID's. Furthermore, the majority (76.2%) of our participants belonged to urban areas. Thus, a study targeting a much broader population may provide results that could be better generalized. Limitation of this study also include the reliability of self-administered questionnaire, which may be partially biased. Finally, our study's response rate was lower than expected; yet it is still comparable to similar published articles [28],[29],[30].

## 5. Conclusion

10.1% and 12.1% of the total population is identified as seriously and moderately unwell (psychologically), respectively. It is very important to deal with psychological issues in the current pandemic. Yao et al. states that online mental health consultation might be more helpful in this pandemic [31]. Our study also shows that a higher knowledge level regarding the disease will help mitigate distress levels. Thus, it is essential to aggressively address mental issues and provide awareness regarding the COVID-19 pandemic to general population. It will help in dealing with the pandemic effectively. Furthermore, periodic surveys must be carried throughout this period to assess the effectiveness of employed methods. Finally, further studies regarding the topic must be carried out to verify our results and find predictive patterns for the general population.
